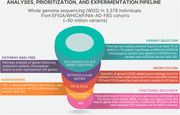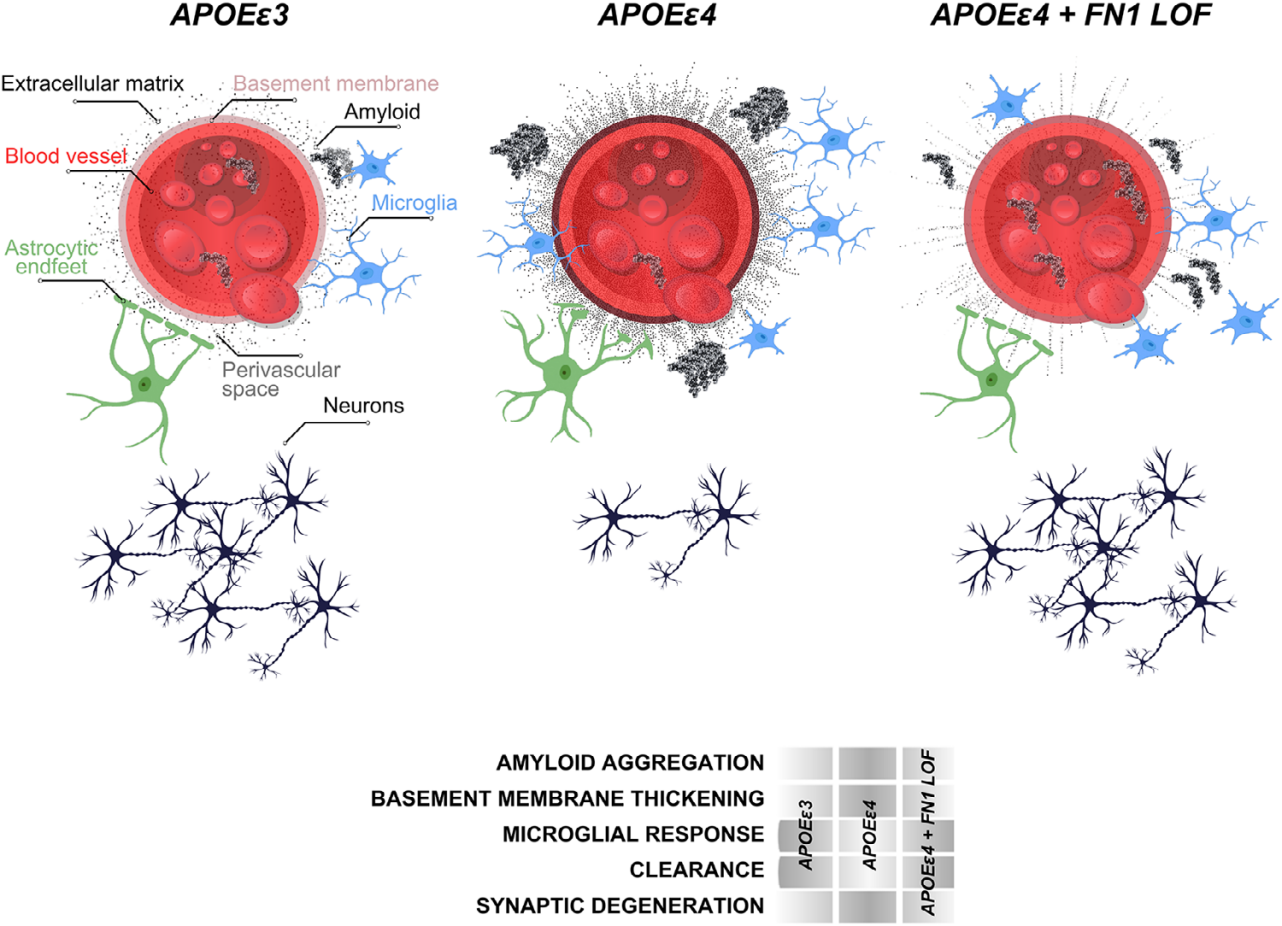# Rare genetic variation in fibronectin 1 (*FN1*) protects against *APOE* ɛ4 in Alzheimer’s Disease

**DOI:** 10.1002/alz.089111

**Published:** 2025-01-03

**Authors:** Prabesh Bhattarai, Tamil Iniyan Gunasekaran, Bengisu Turgutalp Uzrek, Dolly Reyes‐Dumeyer, Dörthe Jülich, Annie J. Lee, Elanur Yilmaz, Huseyin Tayran, Rafael A. Lantigua, Martin Medrano, Diones Rivera Mejia, Patricia Recio, Delaney Flaherty, Clifton L. Dalgard, Tal Nuriel, Nilüfer Ertekin‐Taner, Dennis W. Dickson, Andrew F Teich, Scott Holley, Richard Mayeux, Caghan Kizil, Badri N. Vardarajan

**Affiliations:** ^1^ The Taub Institute for Research on Alzheimer’s Disease and the Aging Brain, New York, NY USA; ^2^ Columbia University Irving Medical Center, New York, NY USA; ^3^ The Gertrude H. Sergievsky Center, College of Physicians and Surgeons, Columbia University, New York, NY USA; ^4^ Columbia University Irving Medical Center, New York City, NY USA; ^5^ Gertrude H. Sergievsky Center, Vagelos College of Physicians & Surgeons, Columbia University, New York, NY USA; ^6^ Department of Neurology, Vagelos College of Physicians and Surgeons, Columbia University, New York, NY USA; ^7^ Taub Institute for Research on Alzheimer’s Disease and the Aging Brain, Columbia University, New York, NY USA; ^8^ Yale University, New Haven, CT USA; ^9^ Department of Neurology, Columbia University Medical Center, New York, NY USA; ^10^ The Taub Institute for Research on Alzheimer’s Disease and The Aging Brain, Columbia University, New York, NY USA; ^11^ Department of Neurology and The Taub Institute for Research on Alzheimer’s Disease and the Aging Brain, Columbia University Irving Medical Center, New York, NY USA; ^12^ School of Medicine, Pontificia Universidad Catolica Madre y Maestra, Santiago Dominican Republic; ^13^ CEDIMAT, Santo Domingo, Santo Domingo Dominican Republic; ^14^ CEDIMAT, Santo Domingo Dominican Republic; ^15^ Uniformed Services University, Bethesda, MD USA; ^16^ Department of Anatomy, Physiology, and Genetics, Uniformed Services University of the Health Sciences, Bethesda, MD USA; ^17^ Department of Cell Biology and Pathology, New York, NY USA; ^18^ Taub Institute for Research on Alzheimer’s disease, New York, NY USA; ^19^ Mayo Clinic, Jacksonville, FL USA; ^20^ Department of Neuroscience, Mayo Clinic, Jacksonville, FL USA; ^21^ Department of Pathology and Cell Biology, New York, NY USA; ^22^ Taub Institute for Research on Alzheimer’s Disease and the Aging Brain, New York, NY USA; ^23^ Columbia University, New York, NY USA; ^24^ Departments of Neurology, Psychiatry, and Epidemiology, Gertrude H. Sergievsky Center, The Taub Institute for Research on Alzheimer’s Disease and the Aging Brain, Vagelos College of Physicians and Surgeons, Columbia University, New York, NY USA; ^25^ The Taub Institute for Research on Alzheimer’s Disease and the Aging Brain, Vagelos College of Physicians & Surgeons, Columbia University, New York, NY USA; ^26^ Department of Neurology, College of Physicians and Surgeons, Columbia University, New York, NY USA; ^27^ Department of Neurology, The New York Presbyterian Hospital, New York, NY USA

## Abstract

**Background:**

*APOEε4* significantly increases the risk of developing Alzheimer’s disease (AD). Cognitively healthy *APOEε4*‐carriers exist, suggesting potential protective mechanisms against *APOEε4*. We hypothesized that some *APOEε4‐*carriers may have genetic variations protecting them from developing *APOEε4*‐mediated AD pathology. We aim to identify these protective genetic variants.

**Methods:**

Whole genome sequencing (WGS) and cerebrospinal fluid (CSF) proteomics were performed from human cohorts to identify potential protective variants segregating exclusively among *APOEε4* carriers. Bioinformatic analyses were performed to select candidate target genes. Immunohistochemistry on postmortem human brain tissues and mouse models expressing human *APOE* variants were performed along with *in‐vivo* functional studies in adult zebrafish AD model.

**Result:**

WGS analyses revealed 510 potential gene variants segregating exclusively among *APOEε4* carriers, which included rare and loss‐of‐function (LOF) variants. Pathway analysis of these genes showed significant enrichment in extracellular matrix (ECM)‐related processes, suggesting protective effects of LOF in ECM proteins. This was further validated by CSF proteome profiling and subsequent analyses in *APOEε4* carriers and non‐carriers. *Fibronectin‐1 (FN1)* and *Collagen‐6A2* (*COL6A2*) were prioritized as candidate target genes for postmortem validation and *in‐vivo* functional studies. FN1 protein was increased in *APOEε4* carriers resulting in thickened ECM at the basement membrane around the blood vessels, potentially impairing pathology‐induced responses such as clearance and immune system activity. This observation is validated in human brains, mouse models and zebrafish model; therefore, the pathological association of FN1 to AD is evolutionarily conserved. Supporting this hypothesis, *in‐vivo* functional study in zebrafish model with LOF mutations in *fn1b* revealed that fibronectin LOF enhanced gliovascular remodeling and microglial activation while reducing astrogliosis, suggesting that pathological accumulation of FN1 could impair toxic protein clearance, which is ameliorated with *FN1* LOF.

**Conclusion:**

The vascular deposition of the ECM components FN1 and COL6A2 are increased in *APOEε4* carriers. Rare variant in *FN1* protect against *APOEε4‐*mediated pathogenesis in AD. We propose a new disease mechanisms and potential therapeutic intervention targets for vascular contribution to dementia to mitigate the risk of developing AD.